# (2,4-Dinitro­phen­yl)(1-methyl-1-nitro­ethyl)diazene

**DOI:** 10.1107/S1600536808034454

**Published:** 2008-10-25

**Authors:** Chunlan Yuan

**Affiliations:** a Department of Chemistry and Chemical Engineering, Baoji College of Arts and Sciences, Baoji 721007, People’s Republic of China

## Abstract

In the title compound, C_9_H_9_N_5_O_6_, the azo group adopts a *trans* conformation and the dihedral angles between the two nitro groups and the benzene ring are 11.6 (3) and 21.3 (3)°.

## Related literature

For general background, see: Hrabie *et al.* (1998 ▶); Batler & Williams (1993 ▶); Murad (1999 ▶); Ignarro (1999 ▶); Wang *et al.*, (2002 ▶); Hrabie & Keefer (2002 ▶). For related compounds, see: Engel (1980 ▶); Katritzky *et al.*, (2002 ▶). For the synthesis, see: Ueno & Umeda (1991 ▶); Zhang *et al.* (1992 ▶). 
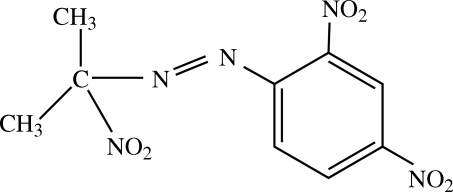


## Experimental

### 

#### Crystal data

C_9_H_9_N_5_O_6_
*M* *_r_* = 283.21
Monoclinic, 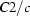

*a* = 13.495 (4) Å
*b* = 12.847 (4) Å
*c* = 14.362 (3) Å
β = 92.18 (3)°
*V* = 2488.1 (12) Å^3^
*Z* = 8
Mo *K*α radiation
μ = 0.13 mm^−1^
*T* = 298 (2) K
0.50 × 0.36 × 0.26 mm

#### Data collection

Siemens P4 diffractometer
Absorption correction: none
9672 measured reflections
2336 independent reflections
1026 reflections with *I* > 2σ(*I*)
*R*_int_ = 0.063
3 standard reflections every 97 reflections intensity decay: 0.8%

#### Refinement

*R*[*F* ^2^ > 2σ(*F* ^2^)] = 0.059
*wR*(*F* ^2^) = 0.204
*S* = 0.84
2336 reflections
183 parameters
H-atom parameters constrained
Δρ_max_ = 0.30 e Å^−3^
Δρ_min_ = −0.21 e Å^−3^

### 

Data collection: *XSCANS* (Siemens, 1996 ▶); cell refinement: *XSCANS*; data reduction: *SHELXTL* (Sheldrick, 2008 ▶); program(s) used to solve structure: *SHELXS97* (Sheldrick, 2008 ▶); program(s) used to refine structure: *SHELXL97* (Sheldrick, 2008 ▶); molecular graphics: *SHELXTL*; software used to prepare material for publication: *SHELXTL*.

## Supplementary Material

Crystal structure: contains datablocks I, global. DOI: 10.1107/S1600536808034454/kp2190sup1.cif

Structure factors: contains datablocks I. DOI: 10.1107/S1600536808034454/kp2190Isup2.hkl

Additional supplementary materials:  crystallographic information; 3D view; checkCIF report

## Figures and Tables

**Fig. 1.**
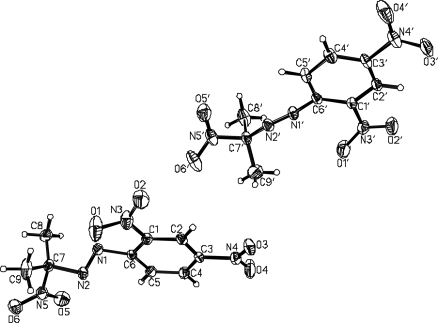
The structure of (I) showing the atom-numbering scheme. Displacement ellipsoids are drawn at the 30% probability level and H atoms are not shown.

**Fig. 2.**
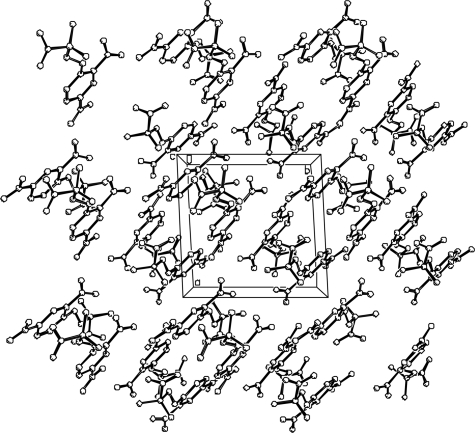
The crystal packing of (I), viewed along the c axis.

## supplementary crystallographic information

### Comment

The important roles of nitric oxide (NO) in atmospheric processes (Hrabie *et al.*, 1998) and in biological events (Batler & Williams, 1993; Murad, 1999; Ignarro, 1999) have known for quite a time. The intensive researches have been directed toward reactions of NO with biological molecules ( Wang *et al.*, 2002; Hrabie & Keefer, 2002). Azoalkanes can be widely used as thermal free radical initiators (Engel, 1980) and some of azoalkanes are specific intermediate in organic synthesis ( Katritzky *et al.*, 2002). In this paper, a new compound of azoalkanes 1-(2,4-dinitrophenyl)azo-2-nitropropylamine, (I), was prepared and its single crystals structure determined.

The structure of (I) (Fig. 1), 1-(2,4-dinitrophenyl)azo-2-nitropropylamine, consists of 2,4-dinitrophenyl and nitropropane linked by an azo group. In three NO_2_, the bonds of O4/N4/O3 are normal (1.202 Å) but the bonds of the N3-O1 and N5-O6 are 1.227 and 1.230 Å, respectively. It is obviously much longer than that of N5-O5(1.180Å),  due to effects of azo double bond. The double N1═N2 connects 2,4-dinitrophenyl and nitropropane.The bond lengths of N2-C7 (1.484Å) is longer than that of N1-C6 (1.433 Å).

### Experimental

A stock solutions were prepared by dissolving 0.5 mol acetone -2,4-dinitrophenylhydrazine in 100 mL dry CH_2_Cl_2_. NO was produced by the reaction of 1 mol H_2_SO_4_ solution with saturated NaNO_2_ aqueous solution. The former was added to the latter, which was stirred under an argon atmosphere. NO was carried by argon and purified by passing it through a series of scrubbing bottles containing 4 M NaOH, distilled water, and CaCl_2_ in turn. All the above bottles were under an argon atmosphere. The purified NO bubbled through a previously degassed stirred stock solution at room temperature for an appropriate time. After the reaction was completed, as indicated by TLC, the reaction mixture was dried with anhydrous MgSO_4_, concentrated in vacuum and purified by column chromatography on silica–gel (200–300 mesh, ethyl acetate–hexane) and the pure title compound was obtained.

### Refinement

(type here to add refinement details)

### Crystal data

**Table:**

C_9_H_9_N_5_O_6_	*F*(000) = 1168
*M**_r_* = 283.21	*D*_x_ = 1.512 Mg m^−^^3^
Monoclinic, *C*2/*c*	Mo *K*α radiation, λ = 0.71073 Å
*a* = 13.495 (4) Å	Cell parameters from 33 reflections
*b* = 12.847 (4) Å	θ = 4.3–13.5°
*c* = 14.362 (3) Å	µ = 0.13 mm^−^^1^
β = 92.18 (3)°	*T* = 298 K
*V* = 2488.1 (12) Å^3^	Prism, yellow
*Z* = 8	0.50 × 0.36 × 0.26 mm

### Data collection

**Table:**

Siemens P4 diffractometer	*R*_int_ = 0.063
Radiation source: fine-focus sealed tube	θ_max_ = 25.6°, θ_min_ = 2.2°
graphite	*h* = −16→11
ω scans	*k* = −11→15
9672 measured reflections	*l* = −17→17
2336 independent reflections	3 standard reflections every 97 reflections
1026 reflections with *I* > 2σ(*I*)	intensity decay: 0.9%

### Refinement

**Table:**

Refinement on *F*^2^	Primary atom site location: structure-invariant direct methods
Least-squares matrix: full	Secondary atom site location: difference Fourier map
*R*[*F*^2^ > 2σ(*F*^2^)] = 0.060	Hydrogen site location: inferred from neighbouring sites
*wR*(*F*^2^) = 0.204	H-atom parameters constrained
*S* = 0.85	*w* = 1/[σ^2^(*F*_o_^2^) + (0.1218*P*)^2^] where *P* = (*F*_o_^2^ + 2*F*_c_^2^)/3
2336 reflections	(Δ/σ)_max_ < 0.001
183 parameters	Δρ_max_ = 0.30 e Å^−^^3^
0 restraints	Δρ_min_ = −0.21 e Å^−^^3^

### Special details

**Table:**

Geometry. All esds (except the esd in the dihedral angle between two l.s. planes) are estimated using the full covariance matrix. The cell esds are taken into account individually in the estimation of esds in distances, angles and torsion angles; correlations between esds in cell parameters are only used when they are defined by crystal symmetry. An approximate (isotropic) treatment of cell esds is used for estimating esds involving l.s. planes.
Refinement. Refinement of F^2^ against ALL reflections. The weighted R-factor wR and goodness of fit S are based on F^2^, conventional R-factors R are based on F, with F set to zero for negative F^2^. The threshold expression of F^2^ > 2sigma(F^2^) is used only for calculating R-factors(gt) etc. and is not relevant to the choice of reflections for refinement. R-factors based on F^2^ are statistically about twice as large as those based on F, and R- factors based on ALL data will be even larger.

### Fractional atomic coordinates and isotropic or equivalent isotropic displacement parameters (Å^2^)

**Table:**

	*x*	*y*	*z*	*U*_iso_*/*U*_eq_	
O1	0.4199 (4)	0.4172 (3)	0.3171 (2)	0.175 (2)	
O2	0.3274 (3)	0.4313 (2)	0.1933 (2)	0.1333 (13)	
O3	0.2968 (2)	0.1231 (3)	0.00469 (19)	0.1178 (11)	
O4	0.2871 (3)	−0.0239 (4)	0.0731 (3)	0.170 (2)	
O5	0.4965 (3)	0.1031 (3)	0.6134 (3)	0.1268 (13)	
O6	0.5905 (3)	0.2187 (3)	0.6742 (2)	0.1470 (15)	
N1	0.3952 (2)	0.2560 (2)	0.42044 (18)	0.0693 (8)	
N2	0.4630 (2)	0.2226 (2)	0.46842 (17)	0.0692 (8)	
N3	0.3672 (3)	0.3804 (3)	0.2542 (3)	0.1072 (13)	
N4	0.3057 (2)	0.0686 (4)	0.0723 (2)	0.0964 (11)	
N5	0.5245 (3)	0.1903 (4)	0.6209 (2)	0.0946 (11)	
C6	0.3831 (2)	0.2084 (3)	0.33028 (19)	0.0553 (8)	
C1	0.3611 (2)	0.2680 (3)	0.2510 (2)	0.0622 (8)	
C2	0.3345 (2)	0.2222 (3)	0.1672 (2)	0.0653 (9)	
H2	0.3181	0.2624	0.1150	0.078*	
C3	0.3330 (2)	0.1171 (3)	0.1629 (2)	0.0669 (9)	
C4	0.3567 (3)	0.0556 (3)	0.2382 (2)	0.0825 (11)	
H4	0.3564	−0.0166	0.2327	0.099*	
C5	0.3809 (2)	0.1023 (3)	0.3226 (2)	0.0698 (10)	
H5	0.3959	0.0613	0.3746	0.084*	
C7	0.4696 (3)	0.2740 (3)	0.5614 (2)	0.0692 (9)	
C9	0.3722 (3)	0.2914 (4)	0.6046 (2)	0.1031 (15)	
H9A	0.3377	0.3472	0.5729	0.155*	
H9B	0.3829	0.3091	0.6691	0.155*	
H9C	0.3331	0.2290	0.5996	0.155*	
C8	0.5338 (5)	0.3655 (4)	0.5534 (3)	0.138 (2)	
H8A	0.5940	0.3458	0.5246	0.207*	
H8B	0.5492	0.3931	0.6144	0.207*	
H8C	0.5001	0.4175	0.5161	0.207*	

### Atomic displacement parameters (Å^2^)

**Table:**

	*U*^11^	*U*^22^	*U*^33^	*U*^12^	*U*^13^	*U*^23^
O4	0.268 (6)	0.114 (3)	0.119 (3)	0.047 (3)	−0.093 (3)	−0.048 (2)
O3	0.131 (3)	0.162 (3)	0.0577 (16)	−0.004 (2)	−0.0226 (16)	−0.0110 (19)
O2	0.202 (4)	0.089 (2)	0.106 (2)	0.006 (2)	−0.039 (2)	0.0264 (19)
O1	0.322 (6)	0.098 (3)	0.101 (2)	−0.070 (3)	−0.063 (3)	0.019 (2)
O5	0.158 (3)	0.100 (2)	0.121 (3)	0.022 (2)	−0.008 (2)	0.023 (2)
O6	0.139 (3)	0.206 (4)	0.092 (2)	0.056 (3)	−0.042 (2)	−0.039 (2)
N1	0.083 (2)	0.0711 (19)	0.0524 (15)	0.0135 (15)	−0.0088 (13)	−0.0039 (14)
N2	0.081 (2)	0.0789 (19)	0.0475 (14)	0.0009 (15)	−0.0033 (13)	−0.0047 (13)
N3	0.163 (4)	0.088 (3)	0.069 (2)	−0.032 (2)	−0.022 (2)	0.014 (2)
N4	0.103 (3)	0.115 (3)	0.070 (2)	0.028 (2)	−0.0264 (18)	−0.025 (2)
N5	0.102 (3)	0.128 (3)	0.0527 (18)	0.028 (2)	−0.0128 (17)	−0.009 (2)
C1	0.067 (2)	0.067 (2)	0.0529 (18)	−0.0033 (16)	0.0005 (14)	0.0048 (16)
C2	0.058 (2)	0.094 (3)	0.0433 (16)	0.0026 (18)	−0.0024 (13)	0.0062 (17)
C3	0.059 (2)	0.090 (3)	0.0503 (18)	0.0127 (18)	−0.0119 (14)	−0.0124 (18)
C4	0.095 (3)	0.077 (2)	0.074 (2)	0.021 (2)	−0.0187 (19)	−0.015 (2)
C5	0.081 (2)	0.072 (2)	0.0556 (19)	0.0187 (18)	−0.0126 (16)	−0.0002 (17)
C6	0.0500 (18)	0.071 (2)	0.0450 (16)	0.0055 (15)	−0.0011 (13)	−0.0048 (15)
C7	0.091 (3)	0.070 (2)	0.0452 (16)	0.0022 (19)	−0.0056 (16)	−0.0030 (16)
C9	0.117 (4)	0.132 (4)	0.060 (2)	0.046 (3)	0.001 (2)	−0.022 (2)
C8	0.200 (6)	0.122 (4)	0.090 (3)	−0.068 (4)	−0.020 (3)	−0.015 (3)

### Geometric parameters (Å, °)

**Table:**

O1—N3	1.227 (4)	C3—C4	1.368 (5)
O2—N3	1.201 (4)	C4—C5	1.381 (4)
O3—N4	1.200 (4)	C4—H4	0.9300
O4—N4	1.215 (5)	C5—H5	0.9300
O5—N5	1.186 (4)	C6—C5	1.368 (5)
O6—N5	1.209 (4)	C6—C1	1.395 (4)
N1—N2	1.203 (3)	C7—C8	1.467 (6)
N1—C6	1.436 (4)	C7—C9	1.493 (5)
N2—C7	1.489 (4)	C9—H9A	0.9600
N3—C1	1.447 (5)	C9—H9B	0.9600
N4—C3	1.478 (4)	C9—H9C	0.9600
N5—C7	1.544 (5)	C8—H8A	0.9600
C1—C2	1.375 (4)	C8—H8B	0.9600
C2—C3	1.351 (5)	C8—H8C	0.9600
C2—H2	0.9300		
			
O3—N4—O4	124.4 (4)	C3—C4—H4	120.6
O3—N4—C3	118.7 (4)	C5—C4—H4	120.6
O4—N4—C3	116.7 (4)	C6—C5—C4	120.5 (3)
O2—N3—O1	124.0 (4)	C6—C5—H5	119.7
O2—N3—C1	119.7 (3)	C4—C5—H5	119.7
O1—N3—C1	116.1 (4)	C8—C7—N2	107.5 (3)
N2—N1—C6	115.0 (3)	C8—C7—C8	116.4 (4)
N1—N2—C7	111.9 (3)	N2—C7—C8	107.5 (3)
O5—N5—O6	124.5 (4)	C8—C7—N5	109.2 (4)
O5—N5—C7	117.6 (3)	N2—C7—N5	101.5 (3)
O6—N5—C7	117.8 (4)	C9—C7—N5	106.6 (3)
C5—C6—C1	118.5 (3)	C7—C9—H9A	109.5
C5—C6—N1	119.9 (3)	C7—C9—H9B	109.5
C1—C6—N1	121.0 (3)	H9A—C9—H9B	109.5
C2—C1—C6	121.3 (3)	C7—C9—H9C	109.5
C2—C1—N3	117.9 (3)	H9A—C9—H9C	109.5
C6—C1—N3	120.8 (3)	H9B—C9—H9C	109.5
C3—C2—C1	118.1 (3)	C7—C8—H8A	109.5
C3—C2—H2	121.0	C7—C8—H8B	109.5
C2—C2—H2	121.0	H8A—C8—H8B	109.5
C2—C3—C4	122.6 (3)	C7—C8—H8C	109.5
C2—C3—N4	117.7 (3)	H8A—C8—H8C	109.5
C4—C3—N4	119.7 (4)	H8B—C8—H8C	109.5
C3—C4—C5	118.9 (4)		
